# An exploration of the perspectives of Dutch adults experiencing a genetic condition on human germline gene editing

**DOI:** 10.1007/s12687-025-00792-5

**Published:** 2025-04-14

**Authors:** S. Jeanne A. N. Arnold, Diewertje Houtman, Isabel R. A. Retel Helmrich, Sander R. Hilberink, Sam R. Riedijk, ‘The DNA dialogues’ Consortium, D. Arets, S. van Baalen, B. van Beers, B. Burgers, M. C. Cornel, C. G. van El, W. P. Geuverink, Y. Geuze-van Horssen, D. Greeven, E. Grob, L. Henneman, E. van Hoek - Burgerhart, M. D. Kasprzak, J. D. Kist, J. W. G. A. Pot, B. Vijlbrief, T. Vrijenhoek, F. H. van der Weij, J. Wiegertjes, M. van Woensel

**Affiliations:** 1 Erasmus Medical Center, Department of Clinical Genetics, Rotterdam, the Netherlands; 2 Research Center Innovations in Care, Rotterdam University of Applied Sciences, Rotterdam, The Netherlands

**Keywords:** Human genome editing, Human germline gene editing, Experiential knowledge, Epistemic injustice, Semi-structured interviews, Focus group study

## Abstract

Views of people with a genetic condition are crucial in deliberations on human germline gene editing (HGGE), but their perspectives are at risk to be devalued due to epistemic injustice and insufficient attention for the possible sensitivities surrounding HGGE. This study set out to explore the perspectives of people dealing with a genetic condition regarding HGGE, and the possible sensitivities surrounding this topic. We conducted a 2-phase qualitative interview (*N* = 29) and focus group study (*N* = 9) on the perspectives of people with or carrying a genetic condition and family members regarding HGGE. Insights from the interviews were used in the organization of the focus groups. We included 38 persons who have experiences with 18 different genetic conditions. Three main themes were identified: personal deliberation on HGGE, HGGE in the context of reproductive decision making and the impact of HGGE on society. Participants stated discussing the topic was controversial, complicated and overwhelming. An informal setting with peer support helped them to process their thoughts and feelings. This study found three main conclusions. First, the perspectives on HGGE are highly influenced by the perception of a genetic condition as a burden or as part of an identity. Secondly, in the deliberation on HGGE, many participants experienced a conflict between accepting a genetic condition and taking action to mitigate potential harm. Thirdly, the subject and object of the deliberation on HGGE mattered: for whom and what for? Moreover, this study has yielded pragmatic recommendations to accommodate sensitivities around discussing HGGE.

**Supplementary Information:**

The online version contains supplementary material available at 10.1007/s12687-025-00792-5.

## Introduction

Genome editing is a rapidly evolving technique, especially with the use of CRISPR/Cas9 (Yang and Chen 2020). This technique has the potential to alter the DNA of human cells, both somatic and germline. In theory, human germline gene editing (HGGE) could remove heritable conditions from the DNA of a human embryo or precursors of oocytes or sperm. Although it is currently legally prohibited in the Netherlands (Overheid 2002), HGGE might become a reproductive intervention to prevent passing on genetic conditions in the future. In this paper, we use the term genetic conditions to refer to both heritable diseases as heritable conditions that can cause disability (as defined by the World Health Organization (WHO) (2023)). HGGE could have far-reaching consequences and raises both ethical and societal questions that require a thorough examination by all stakeholders (Boardman 2020; Commissie Genetische Modificatie & Gezondheidsraad 2017; Coller 2019; Ormond et al. 2019).

Since HGGE has the potential to affect all of humanity, everyone is a stakeholder of HGGE. As stated by the WHO, decisions on HGGE should therefore not only be made by scientists, but should also reflect the views and values of citizens (WHO Expert Advisory Committee on Developing Global Standards for Governance and Oversight of Human Genome Editing, 2021a, b). Views of people with a genetic condition are of special interest as they arguably have a direct interest in HGGE (Kleiderman and Stedman 2020). Moreover, this group is important because they have experiential knowledge. The few studies on patients’ perspectives on HGGE show that due to their lived experience, they indeed have a unique point of view on the potential harms and benefits of HGGE (Beckman et al. 2019; Boardman 2020; Elliott et al. 2022; Genetic Alliance UK & Progress Educational Trust 2017; Geuverink et al. 2023; Hoffman‐Andrews et al. 2019; Hollister et al. 2019; Kaur 2025; Van Dijke et al. 2021). In addition, studies show that people without a disability are incapable of correctly predicting the experiences of people with a disability (Crocker et al. 2015).

However, despite their importance and uniqueness, the perspectives of people with a disability regarding their own life are often devalued, resulting from epistemic injustice (Goering 2008; Scully 2018). Epistemic injustice is a term introduced by Miranda Fricker (2007, p. 1) to refer to forms of injustice that are ‘a wrong done to someone specifically in their capacity as a knower’. An example of this injustice is when someone’s words are thought to be less credible due to prejudices about that person. This injustice has been shown to have far-reaching implications for decision-making that affects people with disabilities (Goering 2008; Kidd and Carel 2017; Scully 2018). Possibly, this also applies to people with a genetic condition who experience a disability.

Furthermore, because people carrying or at risk of genetic conditions have a potential direct interest, they could experience sensitivities when talking about HGGE. HGGE could be seen as a promising technique to prevent passing on their condition in the future, provoking a sense of hope. This hope might however never be fulfilled, since the implementation of HGGE is still uncertain. On the other hand, the potential of erasing their condition from society might feel offensive, evoking a sense of non-acceptance and not-belonging (Boardman 2014; Buchanan 1996; Shakespeare 2005). Thus, HGGE might not be welcome after all.

How people carrying or at risk of genetic conditions experience the sensitivities around discussing the topic of HGGE is to date unknown. To avoid epistemic injustice the current focus group and interview study set out to explore the perspectives of people experiencing a genetic condition on HGGE, and the possible sensitivities surrounding this topic. Moreover, this study aimed to gain insight into how to take these sensitivities into account in the dialogue about HGGE.

## Materials and methods

### Study design

We conducted a 2-phase qualitative study to explore the perspectives of people with a genetic condition and family members regarding HGGE. Our aim was to gain a broad understanding of the perspectives and sensitivities concerning HGGE in this group with experiential knowledge. Individual interviews were conducted first, followed by focus groups that were shaped according to insights derived from the interviews.

#### Interviews

We developed a semi-structured interview guide in co-creation with healthcare professionals including five geneticists, an embryologist and a neurologist (Supplementary material S1). These professionals highlighted the importance of solid knowledge and explanation of HGGE, because of the possibility of creating false hope. We therefore carefully wrote an explanatory introduction to the interviews, emphasizing the uncertainties surrounding the future possibilities of HGGE. The healthcare professionals also questioned the clinical need for HGGE and whether and when HGGE might be preferred over Preimplantation Genetic Testing (PGT). In the interview guide, we therefore included questions on the clinical need for HGGE and on the comparison of HGGE to PGT and Prenatal Diagnosis (PND). During the interview, we used a hypothetical scenario in which HGGE is technically possible and safe to use, without elaborating on possible applications. Towards the end of the interview, we asked the participants how they experienced the interview and about their perceptions of public dialogues on HGGE. The insights derived from the interviews, especially from the experiences of the participants, have led to a number of pragmatic strategies that we used during the focus groups (Supplementary material S2).

#### Focus groups

Concerning the focus groups, we used a variant of the qualitative research method Photovoice in which participants use photos to express their perspectives on a certain topic (Clark-Ibáñez 2004; Jurkowski 2008; Wang and Burris 1997). Before the meeting participants were asked to watch a video about HGGE and to reflect on their feelings regarding HGGE (DNA-dialoog 2020). Then we asked them to send one photo that demonstrated their personal position towards HGGE. During the focus groups we used these photos as conversation starters. Thus by using this Photovoice variant, participants were stimulated to think and feel about their perspective on HGGE prior to the focus group, to offer all of them a way to share (non-verbal) experiences and perspectives including emotions (Clark-Ibáñez 2004; Jurkowski 2008). It also empowered them to set the agenda of the focus group, ensuring that it resonates with their lived realities.

### Recruitment and procedure

#### Interviews

We sent out an invitation for the interviews via email to 34 patient organizations of patients with genetic conditions affiliated with the Dutch Society of Collaborating Parent and Patient Associations (VSOP) (Zicht op Zeldzaam 2025) and asked them to send our invitation to their members (Supplementary material S3). People willing to participate were asked to fill in an online form. A detailed overview of the recruitment process can be found in the supplements (Supplementary material S4). Inclusion criteria were people having or carrying a genetic condition, age above 18 years and Dutch speaking. We were interested in people with experiential knowledge on a genetic condition in general, so we did not focus on any specific genetic condition. After receiving several requests, we decided to include family members of people with a genetic condition too. Before the interview, we asked the participants to sign an Informed Consent Form (ICF) that we sent digitally at least 1 week prior to the interview.

Interviews took place from the 8 th of February till the 15 th of March 2022, either by phone or by video call. Interviews were conducted by one researcher (JA). After 29 interviews had taken place, transcription and data analysis was started to determine data saturation. Thematic saturation was reached after these 29 interviews, with the last 9 interviews not adding new codes. We collected data on the sociodemographic characteristics and the genetic conditions of all participants.

#### Focus groups

Participants were recruited via the VSOP (newsletter and member panel) and via social media (LinkedIn, Facebook). An overview of the recruitment process can be found in the supplements (Supplementary material S4). Participants were informed that they would receive a gift card of 50 euros and that travel expenses would be reimbursed.

The two focus groups (*N* = 9) took place in a private room of a cafe in Rotterdam, The Netherlands, between January and May 2023. This venue was chosen because of its location (accessible by car and public transport), wheelchair-accessibility and pleasant atmosphere. The focus groups were moderated by SH and JA and structured by the photos the participants had sent in preparation. The deliberations were audiotaped and transcribed verbatim. A few weeks after each focus group, we invited the participants to reflect on and evaluate the focus group during a phone call conducted by JA while taking notes that were used in the evaluation.

### Data analysis

Reflexive thematic analysis was performed on all transcripts (Braun and Clarke 2021). Themes were established based on clustering and categorizing the results for each of the research questions and the considerations that surfaced during recruitment. Coding was done manually using ATLAS.ti 22 software (2024). One researcher (JA) coded all interviews and focus groups, by providing each text fragment with one or multiple codes, corresponding to the relevant theme(s). Three encodings were checked by a second researcher (DH). In case of disagreement, the researchers discussed until consensus was reached and changes were implemented accordingly in all encodings. We elaborate on the themes in the results section. In this paper, quotes are included to illustrate the themes. These quotes were translated from Dutch to English after analysis.

## Results

### Study participants

Participants’ demographic and condition-specific characteristics are summarized in Table 1. A majority of the 38 participants were female and atheist. Most participants had a genetic condition (56%). Participants brought in experiences with a wide range of genetic conditions, 18 in total. Approximately 30% of the participants had experiences with Fragile X syndrome.

**Table 1:** Participants’ demographic and condition-specific characteristics^a^

Characteristics		Interviews	Focus groups	Total
Genetic condition				
Genetic condition, *n* (%)	Neurological conditions	20	1	21 (60%)
	*- Fragile X syndrome* *- Von Hippel-Lindau syndrome* *- Neurofibromatosis* *- Batten disease* *- FSHD*^*b*^ *- Bardet Biedl syndrome*	*12* *3* *2* *1* *0* *2*	*0* *0* *0* *0* *1* *0*	*12 (30%)* *3 (8%)* *2 (5%)* *1 (2,5%)* *1 (2,5%)* *2 (5%)*
	Skeletal/Connective tissue conditions	6	2	8 (21%)
	*- Marfan syndrome* *- HME-MO*^*b*^ *- X-linked hypophosphatemia*	*1* *0* *5*	*0* *2* *0*	*1 (2,5%)* *2 (5%)* *5 (13%)*
	Immunodeficiency conditions	3	1	4 (10%)
	*- IgG subclass deficiency* *- X-linked agammaglobulinemia* *- DOCK8 deficiency* *- CVID*^*b*^	*1* *1* *1* *0*	*0* *0* *0* *1*	*1 (2,5%)* *1 (2,5%)* *1 (2,5%)* *1 (2,5%)*
	Metabolic and Cardiovascular conditions	0	2	2 (5%)
	*- Familial Hypercholesterolemia* *- A1AT*^*b*^	*0* *0*	*1* *1*	*1 (2,5%)* *1 (2,5%)*
	Cancer predisposition syndromes	0	1	1 (2,5%)
	*- BRCA1*^*b*^	*0*	*1*	*1 (2,5%)*
	Blood and Hematologic conditions	0	1	1 (2,5%)
	*- Hemophilia*	*0*	*1*	*1 (2,5%)*
	Hearing conditions	0	1	1 (2,5%)
	*- DFNA9*^*b*^	*0*	*1*	*1 (2,5%)*
Role, *n* (%)	Individual with genetic condition	12	9	21 (55%)
	Carrier of genetic condition	1	0	1 (2,5%)
	Carrier and parent of individual with genetic condition	8	0	8 (20%)
	Parent of individual with genetic condition	6	0	6 (15%)
	Sibling of individual with genetic condition	1	0	1 (2,5%)
	Partner of individual with genetic condition	1	0	1 (2,5%)
Sociodemographic characteristics				
Gender, *n* (%)	Female	20	4	25 (64%)
	Male	9	5	14 (36%)
Age, *n* (%)	21–30	3	2	5 (13%)
	31–40	3	0	3 (8%)
	41–50	4	3	7 (18%)
	51–60	7	1	8 (20%)
	61–70	10	2	12 (30%)
	71–80	2	1	3 (8%)
Religion, *n* (%)	Atheist	20	6	26 (68%)
	Non-practicing	1	0	1 (2,5%)
	Christian^*c*^	5	2	7 (18%)
	Unchurched	3	0	3 (8%)
	Anthroposophical	0	1	1 (2,5%)

^*a*^As specified by participants

^b^*HME-MO* Hereditary Multiple Exostoses – Multiple Osteochondromas, *FSHD* Facioscapulohumeral dystrophy, *BRCA1* BReast Cancer gene 1, *A1AT* Alpha- 1 antitrypsin deficiency, *DFNA9* Autosomal dominant deafness- 9

^c^Including Catholic, Protestants, Dutch Reformed Church and non-specified

### Results of interviews and focus groups

The results of the interviews and focus groups are presented together since the themes were mostly overlapping. In the thematic analysis three main themes were identified: personal deliberation on HGGE, HGGE in the context of reproductive decision making and the impact of HGGE on society. The results are sorted accordingly. In the final part of this section, we will present the results of the evaluation. As mentioned in the introduction, we use the term genetic conditions to refer to both heritable diseases as heritable conditions that can cause disability (as defined by the WHO (2023)). However, in this section we use the terms used by the participants.

**Fig. 1:**
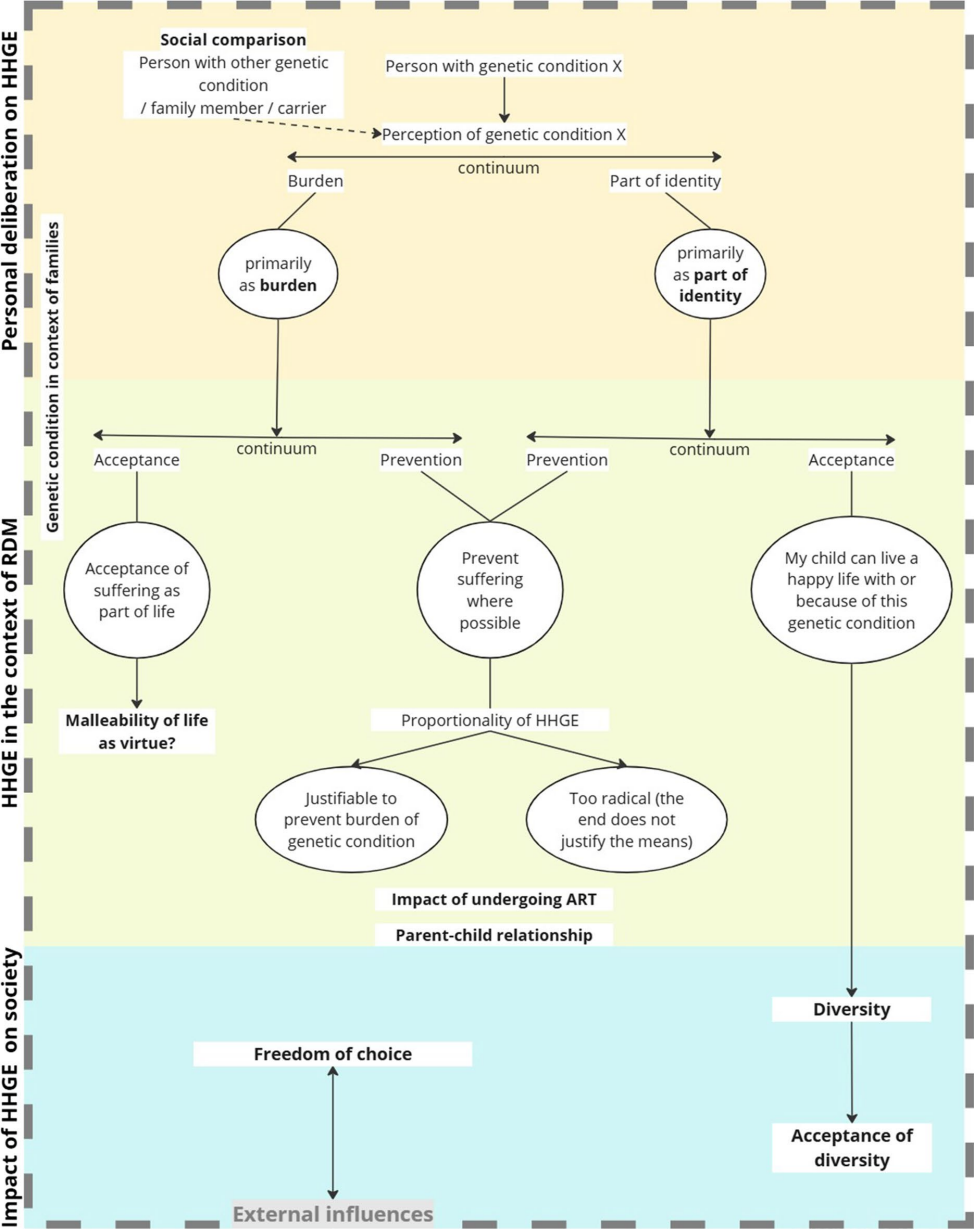
Overview of themes and how they relate to each other within deliberations on HGGE. Themes are displayed in **Bold**. Important to note is that the arrows indicate possible ways of thinking; the deliberations did not yield definitive answers. Instead, the process can be viewed as an ongoing weighing of values, leading to nuanced responses along a continuum. RDM = reproductive decision making. ART = assisted reproductive techniques

Figure 1 provides an overview of the themes and their relationships within the deliberations on HGGE. We identified a pattern in the participants’ considerations. First, they reflected on the role of the genetic condition in their lives, weighing whether it felt more like a burden or part of their identity. When seen as a burden, participants discussed coping with the suffering it caused. When viewed as part of their identity, they considered whether to shield their children from potential suffering or accept the condition as part of their child’s identity. The thought of"preventing suffering where possible"led to reflections on the proportionality of HGGE, while other considerations questioned life’s malleability and valued diversity.

### Personal deliberation on HGGE

#### Burden of genetic condition

During this consideration, some participants expressed sorrow over their symptoms or over the burden of their condition. Participants talked for example about the burden of having regular medical examinations, of hospital visits and of the school or work absenteeism due to their condition.

“With my disease, having all the examinations… Look, they call cancer not without reason a silent killer, you are carrying a time bomb within you, you don’t know it, you don’t feel it, only by screening you know if something is growing or not. That stress… If you could take that away, you should. All these medical procedures and this tension… It’s almost inhumane.” – Individual with a genetic condition (I)

“I’ve been in the hospital so many times… If you could be spared that… You can perform so much better in every way, going to school or whatever… It has been a true obstacle.” – I

[Other participant: do you think this feeling of guilt is part of your condition?]

“[...] I’ve been working on Neuro-Linguistic Programming, a combination of techniques to get to know oneself better etcetera. And having a hereditary condition strongly resurfaces in key moments of my life. The feeling of guilt is part of it... It’s not only the physical part, definitely not” - I

At the same time, participants showed that their attitude to life is highly influenced by their condition and how to deal with it. For example, some participants stated they could deal with setbacks and losing control better than their friends and family due to their lived experience.

“Imagine I didn’t have this illness. That has both positive and negative consequences. Like, yes, the illness has made me very strong in certain aspects... including coping. How do I deal with this? A part of... putting events into perspective. A part of... empathy towards people with illnesses, with medical difficulties. In that sense, it has definitely given me a lot. On the other hand… [...] It can remove so much crap from... from someone’s life. And not just physically. [Other focus group participant: “I can see that it affects you, so…”] Absolutely, also mentally.” - I

#### Genetic condition as part of identity

To some participants, their genetic condition was part of their identity or at least something their identity was highly influenced by. For these participants, the idea that people would prevent children with their genetic condition from being born, made them feel as if they should not be here or as if they were valued less than others.

“If someone would have done that [HGGE] to me, I would feel like being less or inferior than others.” – I.

However, to some, HGGE did feel better than PGT, if it would not require selection.

“HGGE feels much better. If my parents had chosen for PGT, I wasn’t born, although I think my life is totally worth it, I live a very good and happy life, and that opportunity would be denied using PGT.” – I

#### Genetic condition in the context of families

The deliberation on HGGE made participants reflect not only on the impact of their genetic condition on themselves, but also on their family. Some felt guilty, viewing themselves as a burden to relatives or for passing the condition to their children. This guilt was interwoven with sorrow over seeing their child bearing the burden of the genetic condition. Sometimes these feelings were expressed in the form of hope: hope to be able to prevent their offspring from having the condition.

“Well, I have this feeling of guilt, you know there will be difficulties in the future, I see it in my children. I’m starting to go through menopause, I’m getting old and I’m ailing more and more, and that also adds to their responsibility, especially because they know they have the same condition. What’s their vision for the future?” – I

“Looking at my own disease, the idea that I could spare my child that and all the generations to come, that’s a winning lottery ticket, I can’t say otherwise.” – I

“When gene therapy is available for him, he won’t need infusions and that kind of stuff anymore. But that doesn’t mean that his children… No, then I think it’s even more important for him, the HGGE. […] For future generations.” – Carrier of a genetic condition (C), parent of individual with a genetic condition (P)

Conversely, some participants noted that having a genetic condition can strengthen family bonds through shared care for the affected family member. Some participants also mentioned that having the same genetic condition can cause some form of mutual understanding within the parent–child relationship, since both the parent and the child know what it is like to live with a genetic.

“Five years ago, we were really going through a difficult period. And far too often, in my opinion, people want the easy way out with everything, and um, I have to say that through that tough time, it greatly strengthened our relationship, my relationship with my two sons, not just with X [son with genetic condition], tremendously. And it created a unique situation that I’m very happy about and proud of. My little son Y [son without genetic condition] feels the same way. As for X, I can’t get that from him; he doesn’t have the cognitive abilities to express himself on these kinds of matters. So... so I think that when things happen in life and you deal with them, it also strengthens relationships.” – P

#### Social comparison

When talking about HGGE, the subject of the deliberation mattered: whom are we talking about? Participants valued HGGE’s potential differently for different individuals. Participants with a genetic condition often contrasted their own condition or situation with other conditions and situations that they thought of as more severe or worse, weighing the expected degree of suffering up against their own experiences. Because of this, some participants with a genetic condition seemed to be more reluctant in potential applications of HGGE, compared to family members who see a potential to improve the quality of life of their affected family member.

“Do you watch ‘Over mijn lijk’ [a Dutch TV program about adolescents with incurable cancer]? […] I know you’re actually not allowed to compare yourself with people who suffer more, but after watching an episode, I’m always so thankful for my health and life.” – I

“I visit the Erasmus MC [Medical hospital in Rotterdam, the Netherlands] often, and during those visits I see parents with children, that makes me think oh how terrible, how bad, my condition is nothing compared to that, I’m still able to walk, I can have a good day tomorrow… If you could do anything about that, I think that’s even more valuable than for myself.” – I

### HGGE in the context of reproductive decision making

In the context of reproductive decision-making, participants with a genetic condition often made different considerations for their children than for themselves. Some, though content with their own lives, wished to prevent their children from facing the challenges accompanied by having a genetic condition. Others believed that since they lived happily with the condition, their children could too. In case participants wanted to prevent passing on the genetic condition, they thought about the proportionality of HGGE (Fig. 1). What are the potential risks and benefits, and how do these relate?

#### Responsibility of making a reproductive decision

In the hypothetical scenario that HGGE is available to prevent genetic conditions, many participants agreed that this technique could have large consequences for families, especially on reproduction. Due to the availability of HGGE and other assisted reproductive technologies, participants agreed that prospective parents are now forced to make a decision on this matter; doing nothing is a choice too. As a consequence, prospective parents might feel a huge responsibility to make the right reproductive decision.

"Especially because you have that responsibility, I feel that we should also be able to have this conversation, so to speak, because it concerns our lives. It affects my parenthood… and my love for my child, it also has an impact on that."- I

Some participants were afraid that this responsibility was too large to carry. One participant thought this decision was not for the parents to make, since children should be free to develop their own point of view on this matter within their right to an open future.

“If I imagine that there had been an intervention in my DNA while I was still in the womb, then, for me, it would have actually felt like someone was breaking the road there. As I’m driving, I descend to earth, and I think, I’m going to start my path, I’ve picked out a set of genes for that and I’m completely ready for it. And darn it, someone is just cutting and pasting there! So, my own path is already being drastically altered before I even have the chance to tread the path and make it my own.” - I

#### The malleability of life as a virtue?

Many participants questioned the malleability of life as a virtue. On the one hand, participants thought it is good to prevent people from suffering by preventing some genetic conditions from being passed on to next generations. On the other hand, participants doubted if this amount of control over one’s life is actually beneficial. The more control people pretend to have over their lives, the less resilient to unexpected setbacks they will become. One participant also mentioned that having a genetic condition could be seen as one of the many setbacks in life, indicating that it is not the only factor that determines how someone will live his or her life.

“Passing judgment on a life based on the presence of a hereditary condition feels too narrow to me. So, I think there are all sorts of factors that shape a life. A handicap or illness can be just one factor among a multitude of factors. And I don’t believe it’s a crucial determining factor in her [her daughter’s] happiness or unhappiness. Unless she fixates on it blindly. But even that would be her own thing. So, the idea that this one little thing is going to make or break her life, I can’t embrace that. I can’t feel that.” – I

"I think we live in a very engineered society where we want to have control over everything, and, um, I think because I have this, because I have [disease X] and thus don’t have control over it, I feel very distant from that. I have a lot of friends around me who are fortunately completely healthy, but they are all real control freaks, and I am just not like that because I know what it’s like to completely lose control over your life. So, um, I think if we implement this [HGGE], and it becomes an option to determine traits for a future child, um, yes, I think that will only have a negative effect, really. Yes. And, um, I think this will also decrease society’s resilience, because if you are so capable of controlling everything in your life, if you do encounter a setback, you really can’t handle it at all."– I

#### Parent–child relationship

In addition, many participants thought that HGGE will have consequences for the parent–child relationship. For example, some participants were worried the parent’s unconditional love for a child might be affected when parents have too high expectations due to the use of HGGE. However, some participants thought HGGE is better than PGT in this concern, since it may not involve selection.

“Using PGT, you’re actually saying OK, we want a child, but if the embryo is affected, we don’t want it to grow, we abort it, we only let it grow if it’s unaffected. […] Using HGGE, it feels more like, a child is coming, we don’t know how, but it will come.” – I

#### Impact of undergoing assisted reproductive techniques

Moreover, some participants were worried about the impact of the procedure of HGGE on prospective parents; not only physically on the woman who has to undergo the IVF-procedure, but also emotionally. Special attention was given to the value of embryos and what it means to be the “owner” of embryos that are leftovers after the procedure.

“I immediately think of the two healthy embryos, her sisters or brothers, that are in a freezer in Utrecht. And I have no idea what I should do with them. [...] I’ve just had two days of working with a family constellation, [...] and actually, these are all... places in the system that remain empty. They really are. So now I also realize that those potential brothers and sisters, let me just say it, they don’t appear in that [family constellation]. [...] So, that’s basically the conclusion for me, that altering DNA also has far-reaching consequences beyond just the ethical aspect of whether I’m allowed to manipulate DNA or not, whether I can tamper with life, with the structure of life. It carries many disadvantages in terms of emotions, guilt feelings, and indeed the remnants of the process that remain.” - I

### Impact of HGGE on society

Lastly, participants hypothesized what impact HGGE could have on society and societal values.

#### (Acceptance of) diversity

First of all, many participants mentioned the value of diversity. They thought it is important that everyone in society is accepted as they are. When genetic conditions could be prevented by the use of HGGE, participants feared that the acceptance of people with a genetic condition in society would decrease.

“I think it is very important that people are accepted within society. It terrifies me thinking about a society that will judge you because you’re different from others, that you are the exception, and I think that when you can use HGGE for medical reasons, these social aspects won’t get enough attention.” – I

#### Freedom of choice

Furthermore, participants highly valued the freedom of choice to use HGGE for the prospective parents. Almost all participants indicated that this choice is very personal and that they had difficulties with determining this choice for others. Moreover, they stated that this choice should never be influenced by financial pressure, government interference or societal pressure.

“I can’t look in someone’s head, I don’t know what it’s like to be deaf, or to have Down syndrome, so I can’t decide for them.” – I

“And I believe that each person should decide whether they make a decision. Will I alter it or not? And I simply have respect for everyone. Because it’s each individual’s choice, each individual’s perception. And I think you shouldn’t call someone out or attack them for that. No, it’s a conscious choice a parent makes.” – I

### Evaluation

After the interviews, participants explicitly stated the topic is controversial, complicated and overwhelming.

“Geez, that’s quite a story… […] Well, for now, I’m completely empty. I think I’m going home, or actually, I think I will visit the McDonalds for a simple burger, so I’m able to quietly rethink everything we talked about, nice and simple.” – P who participated in an interview

"Yeah, I know plenty who just don’t feel like thinking about something like this. It’s scary and confronting and yucky, and they don’t immediately benefit from it themselves. My own family, for example... way too scary, these kinds of topics. My mother feels guilty about my condition, and talking about it is really hardly feasible, it’s way too scary for her. Even ten years after the diagnosis..."– I who participated in an interview

“[The topic is] definitely burdensome and personal. I was able to incorporate it into the systemic therapy I was doing at the time. It’s really something fundamental, you could say.” – I who participated in a focus group

Participants of the focus groups stated that they looked back on a special and impressive afternoon. They valued the small group setting with peers. Recognition and mutual understanding helped them to learn from each other’s perspective.

“[Due to the focus group I realized that you have to have] respect for everyone, for each person’s standpoint. This subject is so personal, it deals with something so profound, you can’t judge someone else about it."– I who participated in a focus group

"It’s not like my opinion has changed or anything, not that, but I have become more aware of other viewpoints regarding germ line modification. It’s very interesting to hear directly from someone who DOESN’T want it. You do get a kind of perspective development during a dialogue."– I who participated in a focus group

Lastly, participants stated that Photovoice helped them to express their feelings.

"I also found the Photovoice assignment very effective. At first, I really thought it was quite difficult, but later on, an image came to mind quite quickly. It helped me not only to reason but also to feel."– I who participated in a focus group

## Discussion

This study aimed to explore the perspectives of people experiencing a genetic condition regarding HGGE, and the possible sensitivities surrounding this topic. In this exploration, this study found three main conclusions.

First, the perception of identity was very important. Participants to whom their genetic condition is primarily part of their identity, had a different perspective compared to participants to whom their genetic condition is primarily a disease. For the first group, the use of HGGE to eliminate genetic conditions felt like they should not be there or that they are valued less than others, which is also seen in previous literature and links to the expressivist objection argument (Boardman 2014; Buchanan 1996, p. 28; Elliott et al. 2022; Geuverink et al. 2023; Hoffman‐Andrews et al. 2019). To them, the elimination of their condition felt like a step towards a homogenized society without any form of diversity rather than the prevention of suffering. This difference may be explained by the previous finding of Boardman and Hale (2018) that the degree to which people with a disability identify with their impairment, more so than how they valued it, was significant in determining attitudes toward selective reproduction. However, one participant thought the feeling of rejecting her own life by using HGGE was less present in HGGE compared to PGT, because of the absence of discarding affected embryos and the absence of selection. In the literature, this was also stated by experts as a potential advantage of HGGE over PGT (Ranisch 2020).

Secondly, in the deliberation on HGGE, many participants experienced a tension between accepting things as they are and acting to prevent possible harm. For example, some participants thought HGGE would make life too malleable. They feared HGGE would give people a false sense of control over life, making people less resilient to the unexpected. Thinking about a society with HGGE, participants also feared a decrease in acceptance of people with a genetic condition. These fears are shared with participants of previous studies about the public and patient perspective on HGGE (DNA-dialoog 2021; Geuverink et al. 2023; Hoffman‐Andrews et al. 2019; Kaur 2022). Moreover, in the context of reproductive decision making, some participants thought the parent’s unconditional love for a child might be affected as the use of HGGE might inflate what parents expect of their children, which was also mentioned in previous studies (DNA-dialoog 2021; Geuverink et al. 2023). This might be similar to the situation described in another study, in which patients regretted their parents’ search for a cure of their condition, because they would have rather seen that their parents had accepted them fully as they are (Hoffman‐Andrews et al. 2019). In addition, one participant stated that she did not want to deprive her child of its right to an open future, just as the children who participated in a DNA dialogue for children did not want their parents to have changed their DNA (Tijhaar 2020). At the same time, many people with a genetic condition, especially those to whom their genetic condition is primarily a disease as opposed to part of their identity, indicated to sorrow over their disabilities and conveyed to experience feelings of guilt about their fear of being a burden to their relatives. Carriers expressed feeling guilty about passing over their condition to their offspring. These feelings are common in families that are confronted with genetic conditions (McAllister et al. 2007). In some cases, these sensitivities resulted in a sense of hope that others will not have to undergo the same in the future.

Thirdly, the subject and the object of the deliberation on HGGE mattered. For example, participants with a genetic condition often made a different consideration when thinking about themselves compared to thinking about their children. The children involved in the DNA dialogue also held a different perspective on HGGE compared to their parents (Houtman et al. 2021). In addition, some participants with a genetic condition seemed to be more reluctant in potential applications of HGGE, putting their own condition in perspective to more severe conditions, compared to family members who see a potential to improve the quality of life of their affected family member. This is in line with previous literature that showed that professionals, family members and the public seem to structurally underestimate the quality of life of people with a disability, thus expecting a higher clinical need for HGGE than people with a disability might see themselves (Boardman et al. 2018; Crocker et al. 2015). This misperception also exists among people with different disabilities. The mental “hierarchy of impairments” is thus very subjective: every individual ranks impairments or symptoms differently based on their own experiences (Deal 2003; Kaur 2025). Future research should be aware of this. Moreover, it highlights the importance of involving people with a genetic condition directly in the deliberation on HGGE, each person being the expert of his/her own condition. This also implies the importance of autonomy and freedom of choice in decisions on potential applications of HGGE.

### Strengths and limitations

This study has several strengths. Although the number of engagement studies on HGGE has increased over the past years, the number of studies looking at the perspectives of people experiencing a genetic condition on HGGE is still limited (Geuverink et al. 2024). Next to exploring these valuable perspectives, this study has explicitly looked at sensitivities regarding HGGE and talking about HGGE in people experiencing a genetic condition, for example by using a 2-phase design and active reflection by the participants and researchers. This enabled us to propose a number of pragmatic recommendations on how to take these sensitivities into account in the public dialogue about HGGE. Moreover, the inclusion of both adults with a genetic condition, carriers of a genetic condition and their family members, next to the inclusion of various heritable conditions resulted in a wide variety of experiences and therefore a broad understanding of the topic.

Limitations of this study are the following. First, we used a hypothetical scenario in which HGGE is technically possible and safe to use, without elaborating on possible applications or restrictions. This scenario is thus based on a simplification of the technique of HGGE in which not all technical complexities are taken into account. Consequently, participants spoke about HGGE from their interpretation of the technique and their interpretation of what a possible future with HGGE might look like. Therefore, some of the results may not reflect a realistic scenario of how HGGE operates. Nevertheless, perspectives on these possible futures indicate what participants value in decision-making about HGGE (Houtman et al. 2023).

Secondly, given the limited sample size, not all perspectives of people experiencing genetic conditions on HGGE are represented, influencing the generalizability of our data. Moreover, participants were included based on convenience sampling: they were members of patient organizations or followers of one researcher (SH) on social media. The participants were potentially more interested in and positive about HGGE than non-participants. This was endorsed by the fact that one participant revealed that she had encountered a lot of opposing forces discussing HGGE within the peer support group of her patient organization. Besides, most participants were atheists and none of the participants was adherent to another religion than Christianity. This could be relevant, since a previous Dutch study found an influence of religious beliefs on people’s perspectives toward HGGE (Van Baalen et al. 2021).

Lastly, this study provoked a sense of hope in some participants. Some participants seemed to be factual and compared HGGE with other medical techniques that are being developed and that might or might not succeed. However, other participants clearly expected HGGE to be developed, complementing their sense of hope. Since the future of HGGE is uncertain, this hope might never be fulfilled. Some thus may argue this study raises false hope. Defining false hope, however, is difficult, especially in the context of an unpredictable future scenario (Eijkholt 2020). The extent to which this sense of hope is harmful or beneficial, may be a topic of further research.

### Recommendations

Given the insights derived from the interviews and focus groups we would like to propose a number of pragmatic recommendations on how to take the sensitivities experienced by people carrying or at risk of genetic conditions concerning HGGE into account in the public dialogue about HGGE in addition to offering access to their experiential knowledge (see Table 2).

**Table 2:** Recommendations for including people carrying or at risk of genetic conditions in the public dialogue on HGGE

Recommendation	Description
Open invitation	Send out an open invitation, spread by patient organizations, to engage in the deliberation on HGGE that emphasizes that the choice to participate is fully voluntary
Focus group	Create an informal, small group setting with peers. Recognition and mutual understanding helped participants to learn from each other’s perspective
Value experiential knowledge	Value the unique experiential and thus concrete and personal perspective of people who have to do with a genetic condition on HGGE, as opposed to people without this experiential knowledge
Room for doubts and nuances	Use a research method that allows nuanced answers. Personal deliberations on HGGE do not yield definitive answers. Instead, the process can be viewed as an ongoing weighing of values, leading to nuanced responses along a continuum
Room for emotions	Embrace emotions as part of the experiential knowledge
Creative research method	Use a creative research method that helps participants to express their feelings, such as Photovoice
Value the individual perspective	Each person with a genetic condition is the expert of his/her own condition: take their perspective regarding their own condition and quality of life seriously
Relaxing ending	Offer participants something simple and relaxing after the deliberation. Give them a possibility to quietly rethink everything they talked about and to talk things through together

## Conclusion

This study found three main conclusions. First, the perspectives on HGGE are highly influenced by the perception of a genetic condition as a burden or as part of an identity. Secondly, in the deliberation on HGGE, many participants experienced a conflict between accepting a genetic condition and taking action to mitigate potential harm. Thirdly, the subject and object of the deliberation on HGGE mattered: for whom and what for?

## Supplementary Information

Below is the link to the electronic supplementary material.

Supplementary file1 (PDF 278 KB)

## Data Availability

The data that support the findings of this study are not openly available due to reasons of sensitivity and privacy and are available from the corresponding author upon reasonable request. Data are located in controlled access data storage at Erasmus MC.

## References

[CR1] Beckman E, Deuitch N, Michie M, Allyse M, Riggan K, Ormond K (2019) Attitudes toward hypothetical uses of gene-editing technologies in parents of people with autosomal aneuploidies. CRISPR J 2:324–330. 10.1089/crispr.2019.0021 31599684 10.1089/crispr.2019.0021

[CR2] Boardman FK (2014) The expressivist objection to prenatal testing: The experiences of families living with genetic disease. Soc Sci Med 107:18–25. 10.1016/j.socscimed.2014.02.025 24602967 10.1016/j.socscimed.2014.02.025

[CR3] Boardman FK (2020) Human genome editing and the identity politics of genetic disability. J Community Genet 11(2):125–127. 10.1007/s12687-019-00437-4 31489571 10.1007/s12687-019-00437-4 PMC7062953

[CR4] Boardman FK, Hale R (2018) How do genetically disabled adults view selective reproduction? Impairment, identity, and genetic screening. Mol Genet Genomic Med 6(6):941–956. 10.1002/mgg3.463 30196552 10.1002/mgg3.463 PMC6305648

[CR5] Boardman FK, Young P, Warren O, Griffiths F (2018) The role of experiential knowledge within attitudes towards genetic carrier screening: a comparison of people with and without experience of spinal muscular atrophy. Health Expect 21(1):201–211. 10.1111/hex.12602 28703871 10.1111/hex.12602 PMC5750730

[CR6] Braun V, Clarke V (2021) Thematic analysis: a practical guide. SAGE

[CR7] Buchanan A (1996) Choosing who will be disabled: genetic intervention and the morality of inclusion. Soc Philos Policy 13(2):18–46. 10.1017/s0265052500003447 11653297 10.1017/s0265052500003447

[CR8] Clark-Ibáñez M (2004) Framing the social world with photo-elicitation interviews. Am Behav Sci 47:1507–1527. 10.1177/0002764204266236

[CR9] Commissie Genetische Modificatie & Gezondheidsraad (2017) Ingrijpen in het DNA van de mens, Morele en maatschappelijke implicaties van kiembaanmodificatie. Commissie Genetische Modificatie (COGEM). https://www.gezondheidsraad.nl/binaries/gezondheidsraad/documenten/adviezen/2017/03/28/ingrijpen-in-het-dna-van-de-mens/advies-Ingrijpen-in-het-DNA-van-de-mens.pdf. Accessed Nov 2021

[CR10] Coller B (2019) Ethics of human genome editing. Annu Rev Med 27(70):289–305. 10.1146/annurev-med-112717-094629 10.1146/annurev-med-112717-094629 PMC11299715 30691366

[CR11] Crocker TF, Smith JK, Skevington SM (2015) Family and professionals underestimate quality of life across diverse cultures and health conditions: systematic review. J Clin Epidemiol 68(5):584–595. 10.1016/j.jclinepi.2014.12.007 25662007 10.1016/j.jclinepi.2014.12.007

[CR12] Deal M (2003) Disabled people’s attitudes toward other impairment groups: a hierarchy of impairments. Disabil Soc 18:897–910. 10.1080/0968759032000127317

[CR13] DNA-dialoog (2020) DNAdialoog: DNA en zwangerschap - DNA aanpassen met CRISPR, NEMO Kennislink. https://www.youtube.com/watch?v=cbHgb3ppWvw. Accessed Jan 2023

[CR14] DNA-dialoog (2021) Resultaten van de DNA-dialoog - Zo denken Nederlanders over het aanpassen van embryo-DNA. https://dnadialoog.nl/wp-content/uploads/2021/01/Eindrapport-DNA-dialoog-jan-2021.pdf. Accessed Nov 2021.

[CR15] Eijkholt M (2020) Medicine’s collision with false hope: the False Hope Harms (FHH) argument. Bioethics 34(7):703–711. 10.1111/bioe.12731 32134519 10.1111/bioe.12731 PMC7664828

[CR16] Elliott K, Ahlawat N, Beckman ES, Ormond KE (2022) “I wouldn’t want anything that would change who he is.” The relationship between perceptions of identity and attitudes towards hypothetical gene-editing in parents of children with autosomal aneuploidies. SSM - Qual Res Health 2:100151. 10.1016/j.ssmqr.2022.100151

[CR17] Fricker M (2007) Epistemic injustice: power and the ethics of knowing. Oxford University Press. 10.1093/acprof:oso/9780198237907.001.0001

[CR18] Genetic Alliance UK & Progress Educational Trust (2017) Basic understanding of genome editing: the report. https://pet.ultimatedb.net/res/org10/Reports/genomeediting_report.pdf. Accessed 3 Mar 2025

[CR19] Geuverink W, Van El C, Cornel M, Lietaert Peerbolte BJ, Gitsels J, Martin L (2023) Between desire and fear: a qualitative interview study exploring the perspectives of carriers of a genetic condition on human genome editing. Humanit Soc Sci Commun 10(1). 10.1057/s41599-023-01935-0

[CR20] Geuverink WP, Houtman D, Retel Helmrich IRA, Kist JD, Henneman L, Cornel MC, Riedijk SR, The DNA dialogues Consortium (2024) A decade of public engagement regarding human germline gene editing: a systematic scoping review. Eur J Hum Genet. 10.1038/s41431-024-01740-6 10.1038/s41431-024-01740-6 PMC12048525 39609592

[CR21] Goering S (2008) ‘You say you’re happy, but…’: contested quality of life judgments in bioethics and disability studies. Bioeth Inq 5:125–135. 10.1007/s11673-007-9076-z

[CR22] Hoffman-Andrews L, Mazzoni R, Pacione M, Garland-Thomson R, Ormond KE (2019) Attitudes of people with inherited retinal conditions toward gene editing technology. Mol Genet Genomic Med 7(7):e803. 10.1002/mgg3.803 10.1002/mgg3.803 PMC6625087 31190471

[CR23] Hollister BM, Gatter MC, Abdallah KE, Armsby AJ, Buscetta AJ, Byeon YJJ, Cooper KE, Desine S, Persaud A, Ormond KE, Bonham VL (2019) Perspectives of sickle cell disease stakeholders on heritable genome editing. CRISPR J 2(6):441–449. 10.1089/crispr.2019.0034 31742431 10.1089/crispr.2019.0034 PMC6919256

[CR24] Houtman D, Vijlbrief B, Riedijk S (2021) Experts in science communication. EMBO Rep 22(8):e52988. 10.15252/embr.202152988 34269513 10.15252/embr.202152988 PMC8344925

[CR25] Houtman D, Geuverink W, Helmrich IRAR, Vijlbrief B, Cornel M, Riedijk S (2023) “What if” should precede “whether” and “how” in the social conversation around human germline gene editing. J Community Genet 14(4):371–375. 10.1007/s12687-023-00652-0 37326787 10.1007/s12687-023-00652-0 PMC10444910

[CR26] Jurkowski J (2008) Photovoice as participatory action research tool for engaging people with intellectual disabilities in research and program development. Intellect Dev Disabil 46:1–11. 10.1352/0047-6765(2008)46[1:Papart]2.0.Co;2 18271609 10.1352/0047-6765(2008)46[1:PAPART]2.0.CO;2

[CR27] Kaur A (2025) Severity in the genomic age: the significance of lived experience to understandings of severity. Eur J Hum Genet 33(2):176–181. 10.1038/s41431-024-01652-5 38926542 10.1038/s41431-024-01652-5 PMC11840064

[CR28] Kaur A (2022) Human germline genome editing as a potential reproductive choice: an exploratory sociological study in the United Kingdom [Doctoral dissertation, University of Cambridge]. Apollo - University of Cambridge Repository. Cambridge. https://www.repository.cam.ac.uk/handle/1810/343067. Accessed 3 Mar 2025

[CR29] Kidd IJ, Carel H (2017) Epistemic Injustice and Illness. J Appl Philos 34(2):172–190. 10.1111/japp.12172 28303075 10.1111/japp.12172 PMC5324700

[CR30] Kleiderman E, Stedman INK (2020) Human germline genome editing is illegal in Canada, but could it be desirable for some members of the rare disease community? J Community Genet 11(2):129–138. 10.1007/s12687-019-00430-x 31420817 10.1007/s12687-019-00430-x PMC7062950

[CR31] McAllister M, Davies L, Payne K, Nicholls S, Donnai D, MacLeod R (2007) The emotional effects of genetic diseases: implications for clinical genetics. Am J Med Genet A 143A:2651–2661. 10.1002/ajmg.a.32013 17937446 10.1002/ajmg.a.32013

[CR32] Ormond K, Bombard Y, Bonham V, Hoffman-Andrews L, Howard H, Isasi R, Musunuru K, Riggan K, Michie M, Allyse M (2019) The clinical application of gene editing: ethical and social issues. J Pers Med 16(4):337–350. 10.2217/pme-2018-0155 10.2217/pme-2018-0155 31331245

[CR33] Overheid (2002) Embryowet Artikel 24. Overheid. https://wetten.overheid.nl/jci1.3:c:BWBR0013797&paragraaf=6&artikel=24&z=2021-07-01&g=2021-07-01. Accessed 4 Mar 2025

[CR34] Ranisch R (2020) Germline genome editing versus preimplantation genetic diagnosis: is there a case in favour of germline interventions? Bioethics 34(1):60–69. 10.1111/bioe.12635 31448423 10.1111/bioe.12635 PMC6973094

[CR35] Scully JL (2018) From “She would say that, wouldn’t she?” to “Does she take sugar?” epistemic injustice and disability. IJFAB: Int J Fem Approaches Bioethics 11(1):106–124. 10.3138/ijfab.11.1.106

[CR36] Shakespeare T (2005) Solving the disability problem. Whose responsibility? Public Policy Res 12(1):44–48. 10.1111/j.1744-540x.2005.00380.x

[CR37] Tijhaar (2020) In gesprek over DNA in het Sophia Kinderziekenhuis. Reportage DNA-dialoog in het Sophia Kinderziekenhuis*.* NEMO Kennislink. https://www.nemokennislink.nl/publicaties/in-gesprek-over-dna-in-het-sophia-kinderziekenhuis/. Accessed 9 Oct 2024

[CR38] Van Baalen S, Gouman J, Houtman D, Vijlbrief B, Riedijk S, Verhoef P (2021) The DNA-dialogue: a broad societal dialogue about human germline genome editing in the Netherlands. CRISPR J 4(4):616–625 34406039 10.1089/crispr.2021.0057

[CR39] Van Dijke I, Lakeman P, Mathijssen IB, Goddijn M, Cornel MC, Henneman L (2021) How will new genetic technologies, such as gene editing, change reproductive decision-making? Views of high-risk couples. Eur J Hum Genet 29(1):39–50. 10.1038/s41431-020-00706-8 32773775 10.1038/s41431-020-00706-8 PMC7852899

[CR40] Wang C, Burris (1997) Photovoice: concept, methodology, and use for participatory needs assessment. Health Educ Behav 24(3):369–387. http://www.jstor.org/stable/45056507. Accessed 9 Jan 2023 10.1177/109019819702400309 9158980

[CR41] WHO Expert Advisory Committee on Developing Global Standards for Governance and Oversight of Human Genome Editing (2021a) Human genome editing: position paper. World Health Organization. https://iris.who.int/bitstream/handle/10665/342485/9789240030404-eng.pdf?sequence=1. Accessed Nov 2021

[CR42] WHO Expert Advisory Committee on Developing Global Standards for Governance and Oversight of Human Genome Editing (2021b) Human genome editing: recommendations. World Health Organization. https://iris.who.int/bitstream/handle/10665/342486/9789240030381-eng.pdf?sequence=1. Accessed Nov 2021

[CR43] WHO (2023) Disability. https://www.who.int/news-room/fact-sheets/detail/disability-and-health. Accessed 7 Mar 2025

[CR44] Yang L, Chen J (2020) A tale of two moieties: rapidly evolving CRISPR/Cas-based genome editing. Trends Biochem Sci 45(10):874–888. 10.1016/j.tibs.2020.06.003 32616331 10.1016/j.tibs.2020.06.003

[CR45] Zicht op Zeldzaam (2025) Organisaties: Zicht op Zeldzaam. https://zichtopzeldzaam.nl/organisaties/. Accessed Dec 2021

