# Supplementary material for: An exploration of the perspectives of Dutch adults experiencing a genetic condition on human germline gene editing
Source: J Community Genet. 2025 Apr 14;16(5):489–501. doi: 10.1007/s12687-025-00792-5 (PMC12401827; doi:10.1007/s12687-025-00792-5)

Supplementary material

S1. Interview guide

(translated from Dutch to English)

Introduction

Welcome, and thank you for agreeing to participate in this interview. Is it alright with you if I record our conversation? I'd first like to ask a few brief questions about you personally, and then I'll explain more about the content of the interview. If there's any question you'd prefer not to answer, please feel free to let me know, and we will skip it.

Demographic characteristics

- How old are you?
- Do you have a genetic condition yourself?
    - Yes
        - Which genetic condition?
        - Could you tell me how you experience having this condition?
    - No
        - Do you carry a genetic condition?
            - Yes
                - Which genetic condition do you carry?
                - Could you tell me how you experience carrying this condition?
            - No
                - Does a family member has a genetic condition?
                - Could you tell me how you experience having a family member with a genetic condition?
- Do you have children?
    - Yes
        - Did the condition you have or carry play a role before or during the pregnancy?
            - Yes
                - Did you use genetic counseling before or during the pregnancy
                - Did you use genetic testing during conception or during the pregnancy?
                    - Yes
                        - Which technique was used?
    - No
        - Do you have a desire to have children?
- Are you religious?
    - Yes
        - To which religious denomination or philosophical group do you belong?

Background information

This interview is about modifying DNA in embryos.

- Have you ever heard anything about modifying DNA in embryos?
    - ➔ Yes
        - What do you know about modifying DNA in embryos?
            - *background information*
    - ➔ No
        - *->background information*

Background information[1]:
When DNA in embryos is modified, segments of hereditary material are altered in a special type of human cell known as a germ cell. This germ cell will later form the basis of all cells that ultimately make up a human being. Thus, the germ cell is foundational for a new baby. By modifying the germ cell, you also change the future baby. This modification is also passed on to the baby's children, meaning that the DNA modification is continually transmitted to the next generation.

There are various reasons for using this technique. One reason is to remove the predisposition for a genetic condition from the DNA, thereby preventing someone from developing the (hereditary) disease. This is referred to as a medical reason—specifically, the prevention of illness. The future child will not become ill, nor will their children.

Another reason is to 'enhance' a future child, for example, by changing eye color. In this case, it's not about preventing disease but altering a specific trait, such as hair or eye color.

That said, it is important to note that modifying DNA in embryos is not currently being practiced. A global agreement has even been established to refrain from using this technique for now. Researchers first want to explore whether we even want to use this technique. If that is the case, much more research needs to be conducted. Currently, the technique carries many risks, as unintended changes might occur in the DNA at locations that are not intended for modification, which could lead to undesirable consequences. Therefore, it remains uncertain whether this technique will ever be used.

Nevertheless, we would like to discuss it. Before modifying embryo DNA is potentially permitted, we want to know if we want to use the technique and, if so, for what purposes. Since you have experience with a hereditary condition, we are very curious about your thoughts on modifying DNA in embryos. We want to understand your feelings and opinions on the matter. This is the focus of this interview. The interview will last approximately 45-60 minutes.

General questions

- What is your initial reaction when you hear about modifying DNA in embryos?
- What feelings does this technique evoke in you?
- Do you have any idea why you feel this way?
- What are your thoughts on modifying DNA in embryos?
- Do you have any doubts about this technique?
  - If so, could you elaborate on those?
- Do you see any advantages to this technique?
  - If so, could you explain those?

Applications

As I mentioned in the introduction, there are two possible reasons for modifying DNA in embryos. The first reason is medical: to remove the predisposition for a genetic condition in order to prevent someone from developing the (hereditary) disease.

- What is your perspective on modifying DNA in embryos for medical reasons?
- Are there conditions for which you would want to use this technique?
  - If so, could you provide examples?
  - Why would you consider using the technique for these conditions?
- Are there conditions for which you would not want to use the technique?
  - If so, could you provide examples?
  - Why would you not consider using the technique for these conditions?

[1] If the interviewer notices during the conversation that the participant is unfamiliar with certain terms, additional explanations will be provided using the "White Paper on Germline Modification: Terms and Definitions as Used by Our Consortium" from the DNA Dialogue (2021). Publications and appendices related to the report "Results of the DNA Dialogue – How the Dutch View Modifying Embryo DNA" will also be referenced.

- Gray areas
    - For example, glasses
- If the technique becomes available, would you want to use it yourself if you were to have children?
    - Why or why not?

The second reason is non-medical: improving a future child, for example, by changing eye color.

- What is your perspective on modifying DNA in embryos for non-medical reasons?
- Where do you draw the line?
- What do you think is the difference between using this technique for medical versus non-medical reasons (if there is a difference)?

Alternatives

Currently, Pre-implantation Genetic Diagnosis (PGD) and Prenatal Diagnosis can already prevent children from being born with serious conditions.

- Are you familiar with Pre-implantation Genetic Diagnosis (PGD)?

Pre-implantation Genetic Diagnosis (PGD) is also known as embryo selection. It is a special form of IVF, in vitro fertilization, also referred to as test-tube fertilization. In IVF, an egg is fertilized in the laboratory, outside the body, and then placed into the uterus. With PGD, early embryos are examined for serious genetic conditions, and only those embryos that do not show a predisposition are returned to the uterus.

- What do you think are the differences between PGD and modifying DNA in embryos (if there are any)?
- Are you familiar with prenatal diagnosis?

Prenatal Diagnosis involves testing during pregnancy, such as chorionic villus sampling (CVS) and amniocentesis. If this testing indicates that the embryo/baby has a genetic condition, consideration may be given to terminating the pregnancy.

- What do you think are the differences between Prenatal Diagnosis and modifying DNA in embryos (if there are any)?

Consequences

- What do you think is the worst that could happen if DNA in embryos can be modified (worst-case scenario)?
    - In this scenario...
        - Do you think modifying DNA in embryos could have consequences for the relationship between parent and child?
            - Which ones?
        - Do you think modifying DNA in embryos could have consequences for our world and the people living together in a country (for society)?
            - Which ones?
- What do you think is the best that could happen if DNA in embryos can be modified (best-case scenario)?
    - In this scenario...
        - Do you think modifying DNA in embryos could have consequences for the relationship between parent and child?
            - Which ones?
    - Do you think modifying DNA in embryos could have consequences for our world and the people living together in a country (for society)?
        - Which ones?
- In scenarios that fall in between:
    - What are the consequences for the parent-child relationship?

- What are the consequences for society?

Closing

- Is there anything you would like to add? Is there anything we haven't discussed that you would like to talk about?
- How does it feel for you to discuss this topic?
- What emotions did you experience during this conversation?
- Do you have any questions for me?
- Would you like to stay involved in the discussions surrounding the modification of DNA in embryos?

Thank you very much for participating in this interview.

S2. Insights derived from interviews and implications for focus groups

Table X
Insights derived from the interviews combined with implications for focus groups

Insight	Implication
Participants experienced talking about HGGE as overwhelming	Support for potential psychological impact of the dialogue:<br>• Informal setting<br>• Room for reflection and recovery<br>• Peer support
Some interviews could use some extra opinion-questioning/depth	Facilitate an in depth discussion:<br>• Creative qualitative research method<br>• Stimulate participants to ask each other questions
Participants did not directly receive anything in return for participating in an interview	Give something back to participants:<br>• A photographer took an artistic picture of every participant which they received by email

S3. Invitation email to patient organizations

(translated from Dutch to English)

Invitation to patient organizations

Dear Sir/Madam,

My name is JA, I am 22 years old, and I am studying Medicine at Erasmus University in Rotterdam. I am currently working on my master thesis. This research is being conducted in the Department of Clinical Genetics at Erasmus MC, under the supervision of SR and DH. Both were extensively involved in the DNA Dialogue project (www.dnadialoog.nl), which explored how Dutch people feel about modifying embryo DNA.

For my master thesis, I want to further investigate this topic, specifically focusing on the perspectives of patients and carriers of genetic conditions, as well as their healthcare professionals. I would like to interview patients and carriers of genetic conditions, which is why I am reaching out to you. I was wondering if there are patients or carriers of genetic conditions within your organization who might be interested in participating in this research. Perhaps you or someone you know might be interested, or if possible, could an announcement be shared within your organization?

Below, I have prepared a sample email for this purpose. I am also open to suggestions on how the text can be better tailored to the medium through which it will be shared or to the target audience. You can reach me by email or phone at...

I look forward to hearing from you. Thank you in advance for your response.

Sincerely,

JA

Invitation to participants

<u>Subject:</u> Participation in Interview Study on Modifying Embryo DNA

Dear Sir/Madam,

I am writing to you regarding your interest in participating in an interview study on modifying embryo DNA.

My name is JA, and I am studying Medicine at Erasmus University in Rotterdam. I am currently working on my master thesis in the Department of Clinical Genetics at Erasmus MC, under the supervision of SR and DH. Both have been extensively involved in the DNA Dialogue project (www.dnadialoog.nl), which explored how Dutch people feel about modifying embryo DNA.

For my master thesis, I want to delve deeper into this topic, specifically focusing on how patients and carriers of genetic conditions feel about it. In other words, I am very curious about your perspective! Therefore, I would like to interview you regarding the modification of embryo DNA. I am very pleased and grateful that you have shown interest in participating!

<u>Why this research?</u>

By modifying embryo DNA, it could theoretically be possible to remove the predisposition for a genetic condition in the DNA to prevent someone from developing a (hereditary) disease. This application is aimed at individuals with a predisposition for a genetic condition, namely current patients and carriers of genetic conditions, like yourself. We are eager to learn your thoughts on this matter!

It is important to note that this technique is currently not in use, as it is controversial and not yet safe. It is also still unknown whether this technique will ever become available.

<u>What will we do?</u>

We will schedule an appointment for a telephone or online interview with you. During the interview, I will first provide more information about modifying embryo DNA. I will then ask you questions about

your views on the technique and its potential applications and consequences. The interview will last a maximum of one hour.

<u>I want to participate! What now?</u>

Great! In the attachment, you will find the participant information form (PIF). Here you can read everything again at your leisure, and you will find more information regarding the protection of your data. To schedule the interview, we need a few things from you:

- A signed consent form for participants (see attachment).
  - This form is necessary, as we cannot use your interview for our research without it. You can scan the signed form and send it via email to...
  - OR we can send the form by post. You will then receive a return envelope (no stamp needed). For this, we will need your address.
- We would like to know when you are available for the interview and whether you prefer a telephone OR online (via video call) interview.
  - If you choose a telephone interview, please let us know the phone number we can reach you at.
  - If you prefer video calling, we will send you an email invitation for a Microsoft Teams appointment.
  - Note: We can schedule interviews starting from Monday, February 7.
- If you have any questions, please let me know via EMAIL.

We hope to hear from you soon, and thank you in advance for your response!

Kind regards,

On behalf of SR and DH,

JA

S4. Flow diagram of participant inclusion

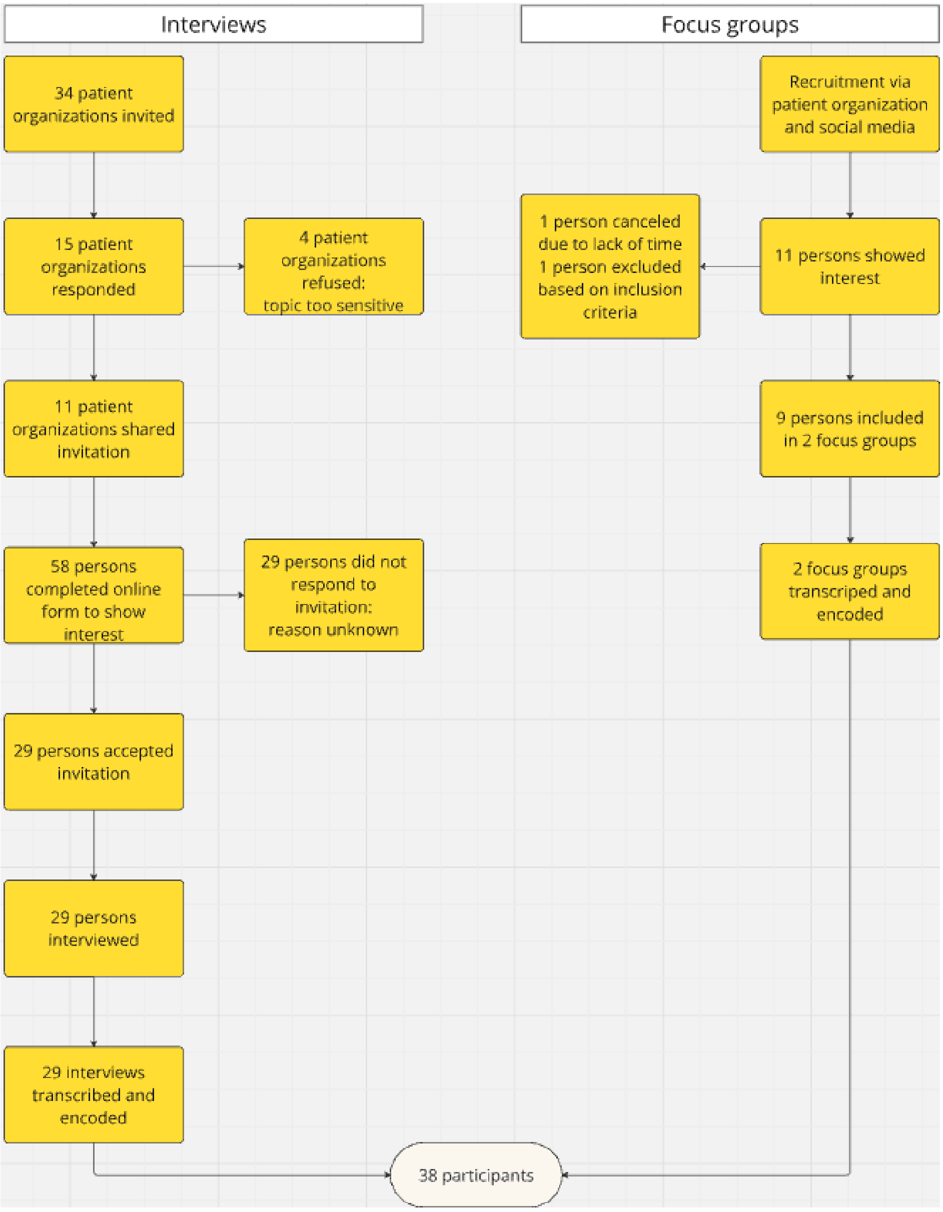

Supplement: Supplementary file 1 — Supplementary file1 (PDF 278 KB) [file 12687_2025_792_MOESM1_ESM.pdf]
